# ESPO AND ACADEMY FOR GERONTOLOGY IN HIGHER EDUCATION SECTION SYMPOSIUM: UTILIZING TECHNOLOGY IN THE ADVANCEMENT OF OLDER ADULT EDUCATION

**DOI:** 10.1093/geroni/igad104.1162

**Published:** 2023-12-21

**Authors:** Yan-Jhu Su, Janelle Fassi, Christine Fruhauf

## Abstract

TThis symposium intends to echo one of the core values of the GSA 2023 Annual Scientific Meeting — empowering all ages. Online lifelong learning has become more common in the 21st century. The speed of technological development far exceeds people’s imagination. The speed at which technology develops is shown to create a widening gap between older and younger populations, known as the digital divide. Introducing technologies into intergenerational learning environments remains an important step to bridging the generational digital divide. In accordance with the AGHE gerontological education competencies, these authors will describe how older adults utilize technology. The first speaker will discuss the effectiveness of a virtual intergenerational program that addressed experiences of ageism for older and younger participants. The second speaker will introduce user-centered design principles and innovative tools that facilitate virtual Information Communications Technology (ICT) support and training for older people with limited abilities in using technology. The third speaker will illustrate concrete practice through international cases from different intergenerational technology integration projects. The fourth speaker will present key elements from the University of Rhode Island (URI) Engaging Generations Cyber-Seniors Program internship and describe student experiences enrolled in the program. The last speaker will discuss the benefits and challenges of technology use among older learners and state the experience of leading older learners using technology in Taiwan. This is a Geriatric Education Interest Group Sponsored Symposium.

